# Relationship between Dubas Bug (*Ommatissus lybicus*) Infestation and the Development of Fungal-Induced Leaf Spots in Date Palms (*Phoenix dactylifera*)

**DOI:** 10.3390/insects14030283

**Published:** 2023-03-14

**Authors:** Salem S. Al-Nabhani, Rethinasamy Velazhahan, Shah Hussain, Suad Al-Raqmi, Maryam Al-Hashmi, Abdullah M. Al-Sadi

**Affiliations:** 1 Directorate General of Agriculture and Livestock Research, Ministry of Agriculture, Fisheries and Water Resources, Muscat 123, Oman; 2 Department of Plant Sciences, College of Agricultural and Marine Sciences, Sultan Qaboos University, Al-Khoud, Muscat 123, Oman

**Keywords:** insect-fungal interaction, ITS, phylogeny, necrosis

## Abstract

**Simple Summary:**

Insect infestation can cause severe loss to the production of agricultural crops in two ways. The first is direct injury to the plant by the feeding insect, which eats the tissues of leaves, stems, fruits, or roots. The second type is indirect damage, in which the insect itself does little or no harm but transmits or influences the infection of bacteria, viruses, or fungi into a crop. We examined the development of fungal infection in date palm leaves following dubas bug infestation. The study provided evidence that dubas bug infestation in date palm leaves facilitated the infection of several fungal species, such as *Alternaria destruens*, *Cladosporium pseudochalastosporoides*, *C. endophyticum*, *Fusarium fujikuroi* species complex, *F. humuli*, *F. microconidium*, *Quambalaria cyanescens*, *Phaeoacremonium krajdenii*, and *P. venezuelense*. This was the first study to report this kind of association between dubas bugs and these fungi in date palms. Future studies should focus on the management of dubas bug infestations to limit the development of other diseases in the host plants.

**Abstract:**

The dubas bug (*Ommatissus lybicus*) (Hemiptera: Tropiduchidae) is a serious pest in date palms in several date-producing countries, including Oman. Infestation results in a severe reduction in yield and a weakening of date palm growth. In addition, egg laying, which causes injuries to date palm leaves, results in the development of necrotic lesions on the leaves. This study aimed at investigating the role of fungi in the development of necrotic leaf spots following dubas bug infestation. Leaf samples developing leaf spot symptoms were collected from dubas-bug-infested leaves, as the leaf spot symptoms were not observed on the non-infested leaves. Isolation from date palm leaves collected from 52 different farms yielded 74 fungal isolates. Molecular identification of the isolates revealed that they belonged to 31 fungal species, 16 genera, and 10 families. Among the isolated fungi, there were five *Alternaria* species, four species each of *Penicillium* and *Fusarium*, three species each of *Cladosporium* and *Phaeoacremonium*, and two species each of *Quambalaria* and *Trichoderma*. Out of the thirty-one fungal species, nine were pathogenic on date palm leaves and induced varying levels of leaf spot symptoms. The pathogenic species were *Alternaria destruens*, *Fusarium fujikuroi* species complex, *F. humuli*, *F. microconidium*, *Cladosporium pseudochalastosporoides*, *C. endophyticum*, *Quambalaria cyanescens*, *Phaeoacremonium krajdenii*, and *P. venezuelense*, which were reported for the first time as leaf spot causal agents in date palms. The study provided novel information on the effect of dubas bug infestation in date palms on the development of fungal infection and associated leaf spot symptoms.

## 1. Introduction

In Oman, date palm (*Phoenix dactylifera* L.) is the most important fruit crop, occupying approximately 50% of the agricultural land of the country. Currently, Oman is the eighth largest producer of dates, producing 374,000 tons [1] from more than 350 different cultivars. Most cultivars are grown for direct human consumption, such as khunaiz cv., Fardh cv., and khellass cv., while others are used as animal feed or for processing. The major production areas of date palm are in Al-Dakhilyah, Al-Batinah, Al-Sharqiya, Al-Dhahira, AL-Buraimi, and Musandam governorates. Date palms have a significant presence in Arabian society. The tree parts have been used for food, feed, shelter, and fuel.

Date palm is affected by many harmful insect pests, attacking all parts of the tree. Approximately 54 insect pests have been reported on date palm trees in Oman [2,3]. Among these insects, the dubas bug (*Ommatissus lybicus*; Hemiptera: Tropiduchidae) has been the most prominent insect affecting date palm trees in Oman since 1962 [4,5]. This bug is extremely devastating to the production of dates, resulting in approximately 28% losses in dates yield in Oman and nearly 50% in Iraq [6,7,8]. Dates of infested palms remain smaller and take longer time to ripen, with a reduction in sugars and sucrose percentages [9]. Many trees have been lost due to infestation by this pest [10]. Other pests of date palms include the red palm weevil *Rhynchophorus ferrugineus* (Olivier) (Coleoptera: Curculionidae), the lesser date moth *Batrachedra amydraula* (Meyrick) (*Lepidoptera: Cosmopterygidae*), the old-world date mite *Oligonychus afrasiaticus* (McGregor) (Prostigmata: Tetranychidae), *Fiorinia* sp. (Hemiptera: Diaspididae), *Parlatoria blanchardi* (Hemiptera: Diaspididae), the red date scale *Phoenicoccus marlatti* (Cockerell) (Homoptera: Phoenicoccidae), *Pseudaspidoproctus hyphaeniacus* (Monophlebidae: Pseudaspidoproctus), *Platypleura arabica* (Mayers) (Hemiptera: Cicadidae), *Arenipases sabella*, the red Palm Mite *Raoiella indica* (Hirst) (*Acari: Tenuipalpidae*), *Vespa orientalis* (*Hymenoptera: Vespidae*), and *Drosophilla melanogaster* (*Diptera: Drosophilidae*) [2,3].

Dubas bug is a hemimetabolous insect that completes its life cycle in three developmental stages: the egg, the nymph, and the adult [11]. It has two reproductive cycles per year, called the spring generation from February to May and the autumn generation from August to November [11,12,13,14,15]. In the spring, females tend to lay eggs on the fifth frond of date palm leaves to provide maximum protection for eggs against heat during the summer season, while in the autumn they tend to lay eggs on the third frond [16]. The life cycle begins with egg laying; the eggs hatch into nymphs and the nymphs molt five times until they reach the adult stage, in 34 to 95 days at 32.5 °C and 20 °C, respectively [17]. The adults are yellowish-brown to greenish in color, with two black spots on their head; the female is larger in size (5–6 mm) and has a strong ovipositor toothed-saw form, while the male is shorter (3–3.5 mm). Furthermore, eggs are mostly embedded inside the date leaf tissue [17]. On average, each female lays about 129–144 eggs [3,18,19,20]. The dubas bug produces honeydew over the leaf, frond, fruits, and surrounding the date palm tree, as a result of sucking sap [21,22]. Similarly, the egg’s oviposition pattern around the fronds, leaves, and leaflets can cause necrotic areas in the tissues of these parts of the plant [23]. However, it is not clear whether the necrotic lesions are due to fungal infections following dubas bug injuries to the leaves.

Insect pests become a serious threat to plant life because insect infestation facilitates fungal infection in plants [24]. Approximately 30 to 40% of losses in crop yield result from pathogen infections following insect infestation [25]. Fungal infection in plants is further spread over a large area by insect vectors [26]. Several fungal species, such as *Alternaria alternata*, *Curvularia lunata*, *Fusarium oxysporum*, *F. proliferatum*, and *Bipolaris oryzae* have been isolated from the adult date palm borer (*Oryctes elegans*) [26]. In mango trees, a secondary infection of *Alternaria* species has been reported following infestation by the mango leaf-gall midge *Procontrinia matteiana* (Kieffer and Cecconi) [27]. *Capnodium* sp., the cause of black sooty mold, has been reported to be associated with the feeding activity of whiteflies and aphids in several crops in Oman, including tomatoes, potatoes, eggplants, chilies, watermelons, sweet melons, chickpeas, and faba beans. Additionally, guava fruit rot, caused by *Alternaria* spp., and inflorescence rot caused by *Fusarium equiseti*, have been reported to be facilitated by insect damage [28].

The study hypothesizes that dubas bug infestation in date palm leaves may favor infection by leaf spot pathogens. The present study was designed to investigate the potential association of fungal species with leaf spots developed in dubas-bug-infested leaves. Knowledge on this subject will clarify whether fungal species play a role in augmenting the damage in date palms caused by the dubas bug, which will be helpful in planning proper management strategies.

## 2. Materials and Methods

### 2.1. Sampling and Isolation of Fungal Isolates from Dubas-Bug-Infested Leaves 

Sampling was carried out at four administrative units (Wilayat) that are famous for date palm production in Oman. Thirteen villages were surveyed: six villages in Izki Wilayat, five in Samail Wilayat, and one each in Al Hamra and Ibri Wilayats. In each village, four date palm farms and fifty date palm trees per farm were surveyed. The details of the sampling sites are given in Table 1. Samples were collected from date palm leaves infested by the dubas bug and developing leaf spot symptoms at the sites of infestation [2,6]. The infestation by dubas bugs was high, approximately 100 insects per leaflet. No spots were observed on leaves not infested by dubas bugs.

For the isolation of fungi, each leaf sample was cut into small sections of a specific size (1 × 1 cm^2^). Each section was surface disinfested with 1% sodium hypochlorite (NaOCl) (Clorox, MAAPICO-NCP, Saudi Arabia) for one minute and then washed in laboratory-produced sterile distilled water for the same period. The sections were dried and then used for the isolation of fungi on 2.5% PDA (potato dextrose agar, Oxoid, UK) medium at a temperature of 27 °C. Emerging fungal colonies were transferred to new Petri dishes, followed by obtaining pure cultures using mycelium tip culture [29]. The pure cultures of the isolated fungi were preserved in slant; i.e., 3.5 mL PDA media with 13% glycerol (Merck KGaA, Darmstadt, Germany).

### 2.2. DNA Extraction, PCR Amplification, and Sequencing

Genomic DNA was extracted from the fungal mycelia using a DNeasy Plant Mini Kit (Qiagen, Valencia, CA, USA) according to the manufacturer’s instructions. The internal transcribed spacer region (ITS1-5.8S-ITS2 = ITS) of nuclear ribosomal DNA was used for the identification of the fungal isolates. Firstly, the ITS1 and ITS4 primer combination was used for the amplification of the ITS region [30]. Polymerase chain reaction (PCR) was performed using PuReTaqTM Ready-To-Go PCR beads (GE Healthcare UK Limited, Buckinghamshire, UK) in a Mastercycler gradient-5331 (Eppendorf, Hamburg, Germany), with 1.0 µL of each primer (10 µmol/L), 22 µL H_2_O, and 1 µL template DNA. The final volume of the PCR mix was 25 µL. PCR conditions were as follows: initial denaturation at 98 °C for 30 s, followed by 35 cycles with denaturation at 98 °C for 10 s, annealing at 55 °C for 30 s, and extension at 72 °C for 30 s; with a final extension for 5 min at 72 °C [29]. The PCR products were purified followed by sequencing using the same primers at Macrogen Inc.^©^ (Seoul, Republic of Korea).

### 2.3. Sequence Alignment and Phylogenetic Analyses

The resulted sequences were trimmed using BioEdit 7.2.5 [31] and were subjected to BLAST analyses to check the sequence similarity against the GenBank sequences in the NCBI database. Sequences of the type of materials of each genus were retrieved from GenBank, and an individual dataset was constructed for each genus for subsequent phylogenetic analysis. Sequences were aligned and assembled using Clustal X 2.1 [32]. For species delimitation, maximum likelihood phylogenetic analysis was performed in RAxML v.7.2.6 [32], using a GTR + G model of evolution with 1000 bootstraps replicated. A bootstrap (BT) proportion of ≥50% was considered significant. Following species delimitation, we combined all the newly generated sequences in a dataset and performed the maximum likelihood analysis in RAxML v.7.2.6 [33] with the same parameters. FigTree 1.4.2 [34] was used for phylogenetic tree visualization, followed by tree annotation using Adobe Illustrator CC2019 (Adobe Inc., San Jose, CA, USA).

### 2.4. Pathogenicity Test

All the isolated fungal species were tested for their pathogenicity on date palm leaflets using detached leaves. The test was conducted on leaflets obtained from 3- to 4-years-old date palms. Whatman No. 1 filter papers were placed in 150 mm Petri dishes and moistened with 50 mg L^−1^ benzimidazole [35]. Then, two 90 mm leaflets were surface disinfected using 1% sodium hypochlorite (NaOCl) for 60 s, washed in sterile distilled water, dried on filter papers, and then placed in a Petri dish in an X shape. Two sets of leaflets were used for each fungal species, one without injury and the other one with superficial injury. The superficial injury was created using a sterilized scalpel, which induced injury on the leaves similar to the ones created by dubas bugs. Mycelial plugs (5 mm) from 2-week-old fungal cultures were placed on the leaflets; two plugs on each leaflet, 50 mm between the plugs. The control leaflets received the same treatment, except using PDA agar plugs instead of mycelial plugs. The Petri dishes were incubated under laboratory room temperature 25 ± 3 °C [35]. There were five replicate Petri dishes for each set of leaves for each fungal species. After 2 weeks of incubation, the development of leaf spots was assessed by measuring the diameter of the area covered with leaf spots. In addition, fungal isolates were re-isolated from the inoculated leaflets. The pathogenicity test was repeated one more time.

### 2.5. Statistical Analysis

Data from the pathogenicity test were statistically analyzed using the Statistical Analysis System, SAS (SAS Institute, Inc., Cary, NC, USA). The separation of means was based on Tukey’s Studentized range test. Standard deviations were also determined for all means.

## 3. Results

### 3.1. Fungal Identification

**Figure 1:**
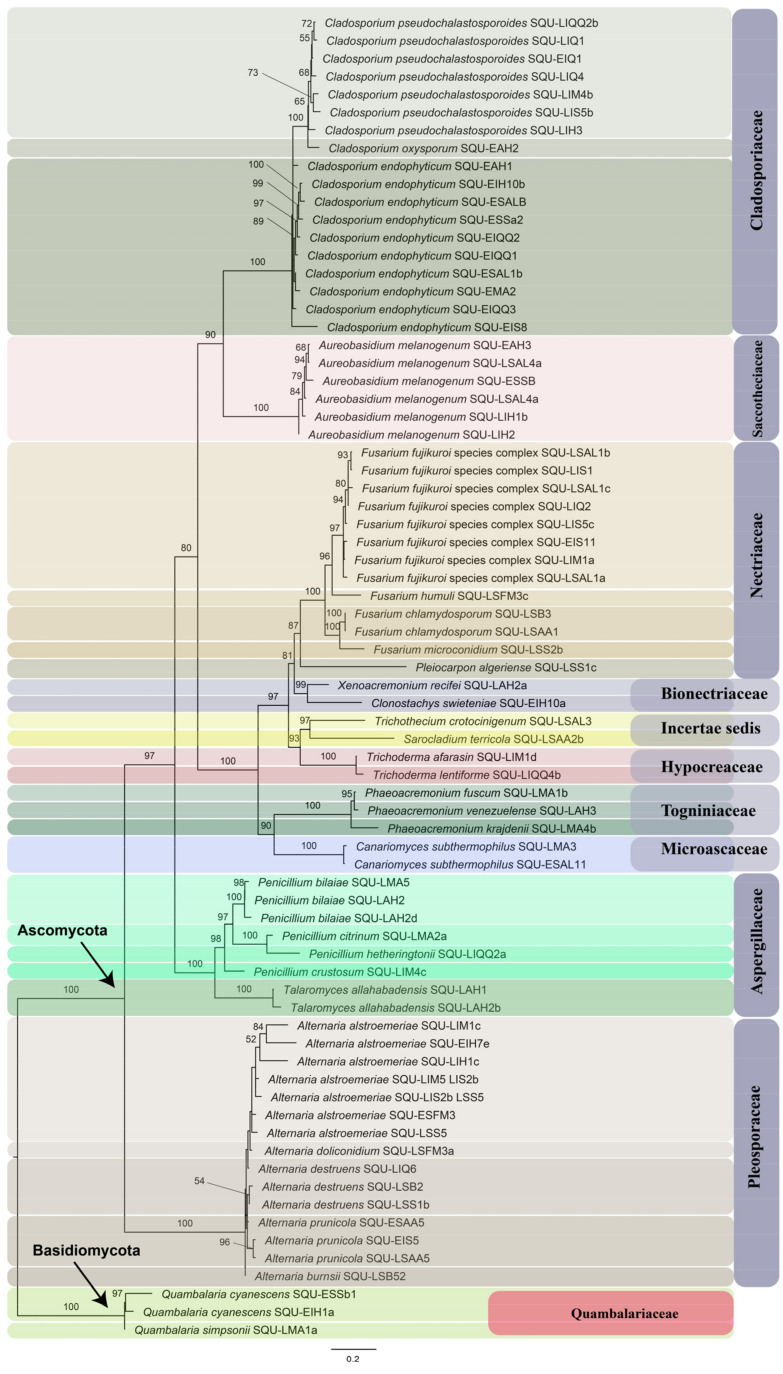
Maximum likelihood phylogenetic tree of individual fungal isolates obtained in this study. The phylogeny is based on the ITS sequences generated during this study, each species of individual genus is highlighted in similar color shade. The taxa represent nine families of Ascomycota and one of Basidiomycota. Two genera *Sarocladium* and *Trichothecium* are in *Incertae sedis* following the modern classification system of fungi [36]. The numbers on branches are the bootstrap values.

The isolated fungi were identified using the ITS barcode. A total of 74 fungal isolates were recovered from the dubas-bug-infested leaves, representing 31 fungal species belonging to 16 genera of 10 fungal families. The maximum likelihood phylogenetic tree is depicted in Figure 1, where each species is highlighted in a specific color shade with their respective family.

### 3.2. Isolation of Fungal Isolates from Dubas Bug-Infested Leaves 

Among the sixteen genera, *Alternaria* was the highly diverse genus with five species; followed by *Penicillium* with four species; *Cladosporium*, *Fusarium*, *Phaeoacremonium* each with three species; and *Quambalaria*, *Trichoderma* each with two species. The remaining genera had a single species each: *Aureobasidium melanogenum*, *Canariomyces subthermophilus*, *Clonostachys swieteniae*, *Pleiocarpon algeriense*, *Sarocladium terricola*, *Talaromyces allahabadensis*, *Trichothecium crotocinigenum*, and *Xenoacremonium recifei*. The most frequently isolated species was *Cladosporium endophyticum* with ten isolates, followed by *Fusarium fujikuroi* with eight isolates, *Alternaria alstroemeriae* and *Cladosporium pseudochalastosporoides* each with seven, *Aureobasidium melanogenum* with six, *Alternaria destruens*, *A. prunicola*, and *Penicillium bilaiae* each with three isolates. The remaining nineteen fungal species were represented by one isolate each.

The highest number of fungal species was recorded from date palm plantations of the villages Alhajer and Mesfat alabriyn, with seven species each, followed by Sifalat with six species, while the lowest number of species was reported from Qarrot South (3). The details are presented in Table 2.

### 3.3. Pathogenicity Test

**Figure 2:**
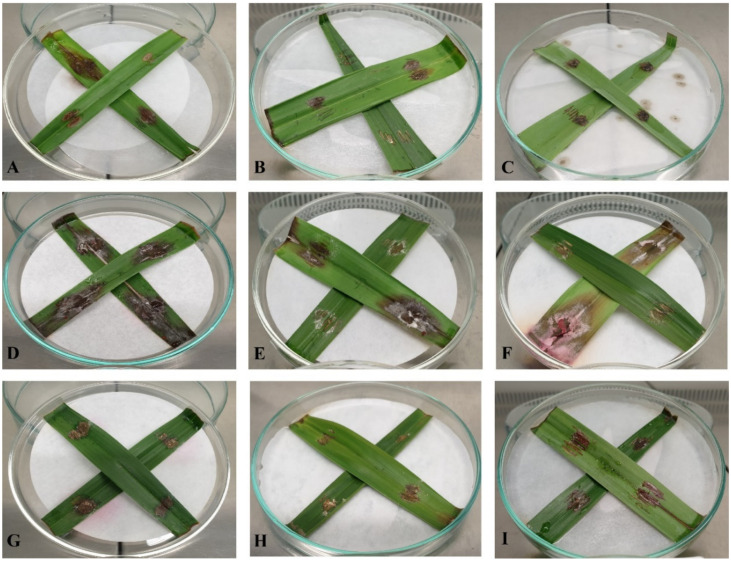
Pathogenic results of detached leaf assays in leaflets of date palm inoculated with (**A**) *Alternaria destruens* SQU-LSS1b, (**B**) *Cladosporium endophyticum* SQU-EIQQ1, (**C**) *Cladosporium pseudochalastosporoides* SQU-LIQ4, (**D**) *Fusarium fujikuroi* species complex SQU-LIS1, (**E**) *Fusarium humuli* SQU-LSFM3c, (**F**) *Fusarium microconidium* SQU-LSS2b, (**G**) *Phaeoacremonium krajdenii* SQU-LMA4b, (**H**) *Phaeoacremonium venezuelense* SQU-LAH3, and (**I**) *Quambalaria cyanescens* SQU-EIH1a.

Pathogenicity tests of the 31 fungal species showed varying levels of leaf spot development. Nine fungal species induced leaf spot symptoms on the wounded leaflets, while twenty-two species resulted in no symptoms. The nine species that induced symptoms are shown in Table 3 and Figure 2. They resulted in brown to black leaf spots that ranged in size from 3.4–9.0 mm within two weeks of inoculation. The highest leaf spot severity was induced by *Phaeoacremonium krajdenii* (9.0 mm), followed by the *Fusarium fujikuroi* species complex (8.2 mm), *F. humuli* (8.2 mm), and *F. microconidium* (8.1 mm). The least disease severity was caused by *Phaeoacremonium venezuelense* (3.4 mm), followed by *Quambalaria cyanescens* (5.6 mm). However, no symptom development was observed on the uninjured leaves, or the control leaves for up to two weeks of incubation.

## 4. Discussion

Dubas bug infestation in date palms has always been found to be associated with the development of necrotic areas (leaf spots) on the affected leaves. This study aimed at investigating the role of fungal species in the development of leaf spots following dubas bug infestation. A total of 31 fungal species, belonging to 16 fungal genera, were isolated from necrotic areas on date palm leaves infested by dubas bugs. Ascomycota was represented by 15 genera, and Basidiomycota was represented by the genus *Quambalaria* J.A. Simpson.

Inoculation of date palm leaves revealed that nine out of thirty-one fungal species induced leaf spot symptoms on the injured leaves. The injury, which was mechanically induced on the leaves, was similar to the ones created by dubas bugs, in order to understand the effect of this injury on the infection by fungi. No symptoms developed on the non-injured leaves. This may suggest that these fungal species cannot penetrate the leaf tissue of a date palm by themselves, and that they need a source of injury. This provides evidence that the fungal species cause leaf spot symptoms following injuries caused by dubas bugs when they insert eggs into the leaves. Insects are known to play an important role in infections by plant pathogenic fungi, which usually happens by facilitating entry through wounds created by insects [37,38,39]. *Fusarium* spp., causing root rot disease in forage legumes, was found to be common in clovers infested with clover root curculio *Sitona hispidulus* F. (Coleoptera: Curculionidae) [38,40,41]. Maize roots chewed by the worm of western corn root (*Diabrotica virgifera virgifera* Le Conte; Coleoptera: Chrysomelidae) help in the colonization by *Fusarium verticillioides* (Saccardo) Nirenberg [42]. *Fusarium* and *Pythium* spp. have been reported to be transported by the fungus gnat larvae (*Bradysia* spp.; Diptera: Sciaridae) in legumes such as alfalfa and soybean [43,44]. *Phytophthora* spp. were isolated from the root tissues of citrus injured by the weevil *Diaprepes abbreviatus* L. (Coleoptera: Curculionidae) [45,46].

Our findings showed that *Alternaria destruens*, *Fusarium fujikuroi* species complex, *F. humuli*, *F. microconidium*, *Cladosporium pseudochalastosporoides*, *C. endophyticum*, *Quambalaria cyanescens*, *Phaeoacremonium krajdenii*, and *P. venezuelense* induced varying levels of leaf spot symptoms on date palm leaves. *Alternaria* is a genus with several species causing leaf spots on different plants. *Alternaria destruens* has been reported to be associated with leaf spots on *Lingustrum sinense* in China [47]. Similarly, in Iran, *A. destruens* has been reported to be associated with black spot disease in cabbage [48]. This is the first record of the association of *A. destruens* with date palm leaf spot.

*Fusarium* is a widely distributed pathogen in different crops in different parts of the world. *Fusarium fujikuroi* is a pathogen of rice seeds [49], maize sheath rot [50], round spot disease of *Zanthoxylum armatum* [51], and brown leaf spot on kiwifruit [52]. *Fusarium humuli* has been reported to cause wilt in yams in China [53], while *F. microconidium* is a recently described species with unknown origin and unknown substrate, with an ex-type culture located in the Centraal Bureau voor Schimmelcultures (CBS) culture collection (CBS 119843 = MRC 8391) [54]. This is the first record of the association of the *F. fujikuroi* complex, *F. humuli*, and *F. microconidium* with date palm leaf spot.

Our findings showed that two *Cladosporium* species are pathogenic on date palm leaves, which are *C. pseudochalastosporoides* and *C. endophyticum*. There have been limited studies on the pathogenicity of *C. pseudochalastosporoides* and *C. endophyticum*. This is the first report of an association of these *Cladosporium* species with date palm leaf spot.

*Quambalaria cyanescens* has been reported to be associated with leaf spot and branch and shoot blight in Iranian grapevine [55]. *Quambalaria* spp. have also been reported to be associated with trunk necrosis and canker and leaf spot symptoms in *Eucalyptus* and *Corymbia* plants [56]. This is the first record of the association of *Q. cyanescens* with date palm leaf spot.

*Phaeoacremonium krajdenii* has been reported to be associated with chlorotic and black spots in xylem vessels and stunted leaves on grapevines (*Vitis vinifera*) [57,58,59,60,61,62]. *Phaeoacremonium krajdenii* has also been reported in *Prunus* fruit tree orchards, such as cherry, peach, and apricot, with several symptoms including defoliation, gummosis, and dieback of branches [63]. Other species, *P. venezuelense*, have been reported to cause a decline and dieback in *Vitis vinifera* [57,58,64], a dieback of branches, and shoots and leaf discoloration and black spot in branches of apricot trees (*Prunus armeniaca*) in Spain [65], and dieback and gummosis on branches of *Calligonum amoenum* in Iran [66]. This is the first record of the association of *P. krajdenii* and *P. venezuelense* with date palm leaf spot.

The present study provides evidence that dubas bug infestation in date palm leaves facilitates infection by *Alternaria destruens*, *Fusarium fujikuroi* species complex, *F. humuli*, *F. microconidium*, *Cladosporium pseudochalastosporoides*, *C. endophyticum*, *Quambalaria cyanescens*, *Phaeoacremonium krajdenii*, and *P. venezuelense*, which results in leaf spot symptoms. The infection by fungi is largely because of the injuries created by the dubas bug on the leaves. This is the first study reporting this kind of association between dubas bug and these fungi. Future studies should focus on the management of dubas bug infestation to limit the development of other associated diseases.

## Figures and Tables

**Table 1:** Sample collection of date palm leaves infested with dubas bug in different administrative units in Oman.

Wilayat	Village	No. of Farms	No. of Leaflets/Tree	Coordinates
Izki	Saima	4	25	22.96179, 57.91549
	Qarrot South	4	25	22.98207, 57.78078
	Muqazzah	4	25	23.01378, 57.87333
	Al humaydah	4	25	22.90664, 57.91260
	Al qariyatain	4	25	22.93293, 57.76855
	Al kharmah	4	25	22.84934, 57.95692
Samail	Sifalat	4	25	23.31527, 58.02095
	Alayat	4	25	23.30303, 57.98520
	Alaainh	4	25	23.29954, 58.06131
	Bory	4	25	23.16340, 57.92657
	falaj almaraghah	4	25	23.17980, 57.90918
Ibri	Alhajer	4	25	23,22415, 56.47231
Al Hamra	Mesfat alabriyn	4	25	23.14048, 57.31255

**Table 2:** Fungal species isolated from the infected leaves of date palm collected from different villages in Oman.

Wilayat	Wilayat	Fungi Isolated from Leaflets
Al Hamra	Mesfat alabriyn	*Canariomyces subthermophilus* (Mouch.) X. Wei Wang and Houbraken
		*Cladosporium endophyticum* Tibpromma and K.D. Hyde
		*Quambalaria simpsonii* Cheew. and Crous
		*Penicillium bilaiae* Kucey
		*Penicillium citrinum* Thom
		*Phaeoacremonium fuscum* L. Mostert, Damm and Crous
		*Phaeoacremonium krajdenii* L. Mostert, Summerb. and Crous
Izki	Al-Qariyatain	*Cladosporium pseudochalastosporoides* Bensch, Crous and Braun
		*Penicillium hetheringtonii* Houbraken, Frisvad and Samson
		*Trichoderma lentiforme* (Rehm) P. Chaverri, Samuels and Rocha
	Qarrot South	*Alternaria destruens* Simmons
		*Cladosporium pseudochalastosporoides* Bensch, Crous and Braun
		*Cladosporium endophyticum* Tibpromma and Hyde
		*Fusarium fujikuroi* Nirenberg species complex
	Muqazzah	*Alternaria alstroemeriae* Simmons and Hill
		*Cladosporium pseudochalastosporoides* Bensch, Crous and Braun
		*Fusarium fujikuroi* Nirenberg species complex
		*Penicillium crustosum* Thom
		*Trichoderma afarasin* Chaverri and Rocha
	Saima	*Alternaria alstroemeriae* Simmons and Hill
		*Alternaria prunicola* Chethana, Yan, Li and Hyde
		*Cladosporium pseudochalastosporoides* Bensch, Crous and Braun
		*Cladosporium endophyticum* Tibpromma and Hyde
		*Fusarium fujikuroi* Nirenberg species complex
	Al-Humaydah	*Alternaria alstroemeriae* Simmons and Hill
		*Aureobasidium melanogenum* (Hermanides-Nijhof) Zalar P., Gostincar, Gunde-Cimerman
		*Cladosporium pseudochalastosporoides* Bensch, Crous and Braun
		*Cladosporium endophyticum* Tibpromma and Hyde
		*Clonostachys swieteniae* Perera, Jones and Hyde
		*Quambalaria cyanescens* (de Hoog and de Vries) de Beer, Begerow and Bauer
Ibri	Alhajer	*Aureobasidium melanogenum* (Hermanides-Nijhof) Zalar P., Gostincar, Gunde-Cimerman
		*Cladosporium oxysporum* Berk. and Curtis
		*Cladosporium endophyticum* Tibpromma and Hyde
		*Penicillium bilaiae* Kucey
		*Phaeoacremonium venezuelense* Mostert, Summerb. and Crous
		*Xenoacremonium recifei* (Leao and Lobo) Lombard and Crous
		*Talaromyces allahabadensis* (Mehrotra and Kumar) Samson, Yilmaz and Frisvad
Samail	Alaainh	*Alternaria prunicola* Chethana, Yan, Li and Hyde
		*Fusarium chlamydosporum* Wollenweber and Reinking
		*Sarocladium terricola* (Mill., Giddens and Foster) Giraldo, Gené and Guarro
	Alayate	*Canariomyces subthermophilus (Mouch.) Wei Wang and Houbraken*
		*Cladosporium endophyticum* Tibpromma and Hyde
		*Aureobasidium melanogenum* (Hermanides-Nijhof) Zalar P., Gostincar, Gunde-Cimerman
		*Fusarium fujikuroi* Nirenberg species complex
		*Trichothecium crotocinigenum* (Schol-Schwarz) Summerb., Seifert and Schroers
	Sifalat	*Alternaria alstroemeriae* Simmons and Hill
		*Alternaria destruens* Simmons
		*Aureobasidium melanogenum* (Hermanides-Nijhof) Zalar, Gostincar, Gunde-Cimerman
		*Cladosporium endophyticum* Tibpromma and Hyde
		*Fusarium microconidium* Lombard and Crous
		*Quambalaria cyanescens* (de Hoog and de Vries) de Beer, Begerow and Bauer
	Falaj almaraghah	*Alternaria alstroemeriae* Simmons and Hill
		*Alternaria doliconidium* Li, Camporesi and Hyde
		*Fusarium humuli Wang, Qian Chen and Cai*
		*Pleiocarpon algeriense* Aigoun-Mouhous, Cabral and Berraf-Tebbal
	Bory	*Alternaria destruens* Simmons
		*Alternaria burnsii* Uppal, Patel and Kamat
		*Fusarium chlamydosporum* Wollenw. and Reinking

**Table 3:** Pathogenicity test of fungal species on date palm leaves.

Fungal Species *	Mean Diameter of Leaf Spot(mm ± SD) **
*Phaeoacremonium krajdenii*	9.0 ± 1.2 a
*Fusarium fujikuroi* species complex	8.2 ± 1.9 a
*Fusarium humuli*	8.2 ± 1.5 a
*Fusarium microconidium*	8.1 ± 1.8 a
*Alternaria destruens*	7.9 ± 1.7 ab
*Cladosporium pseudochalastosporoides*	7.8 ± 1.4 ab
*Cladosporium endophyticum*	5.9 ± 1.3 bc
*Quambalaria cyanescens*	5.6 ± 1.2 c
*Phaeoacremonium venezuelense*	3.4 ± 2.0 d

* Data is shown for the nine fungal species that resulted in leaf spot symptoms. The other 22 species (Table 1) did not cause any symptoms on the inoculated leaflets. ** The values indicate the average of 20 leaf spot measurements for each fungal isolate ± Standard Deviation (SD). Means with the same latter are not significantly different from each other at *p* < 0.05.

## Data Availability

The sequence data presented in this study are openly available in GenBank (https://www.ncbi.nlm.nih.gov/genbank/) (accessed on 6 March 2023).
